# Living roots magnify the response of soil organic carbon decomposition to temperature in temperate grassland

**DOI:** 10.1111/gcb.12784

**Published:** 2014-12-23

**Authors:** Paul W Hill, Mark H Garnett, John Farrar, Zafar Iqbal, Muhammad Khalid, Nawaf Soleman, Davey L Jones

**Affiliations:** 1 School of Environment, Natural Resources and Geography, Bangor UniversityBangor, Gwynedd, LL57 2UW, UK; 2 NERC Radiocarbon Facility, Scottish Enterprise Technology ParkEast Kilbride, G75 0QF, UK; 3 School of Biological Sciences, Bangor UniversityBangor, Gwynedd, LL57 2UW, UK; 4 Nuclear Institute for Agriculture and BiologyFaisalabad, Pakistan; 5 Institute of Soil and Environmental Sciences, University of AgricultureFaisalabad, Pakistan

**Keywords:** acclimation, carbon cycle, climate change, mineralisation, priming, soil organic matter, soil respiration, SOM

## Abstract

Increasing atmospheric carbon dioxide (CO_2_) concentration is both a strong driver of primary productivity and widely believed to be the principal cause of recent increases in global temperature. Soils are the largest store of the world's terrestrial C. Consequently, many investigations have attempted to mechanistically understand how microbial mineralisation of soil organic carbon (SOC) to CO_2_ will be affected by projected increases in temperature. Most have attempted this in the absence of plants as the flux of CO_2_ from root and rhizomicrobial respiration in intact plant-soil systems confounds interpretation of measurements. We compared the effect of a small increase in temperature on respiration from soils without recent plant C with the effect on intact grass swards. We found that for 48 weeks, before acclimation occurred, an experimental 3 °C increase in sward temperature gave rise to a 50% increase in below ground respiration (ca. 0.4 kg C m^−2^; *Q*_10_ = 3.5), whereas mineralisation of older SOC without plants increased with a *Q*_10_ of only 1.7 when subject to increases in ambient soil temperature. Subsequent ^14^C dating of respired CO_2_ indicated that the presence of plants in swards more than doubled the effect of warming on the rate of mineralisation of SOC with an estimated mean C age of ca. 8 years or older relative to incubated soils without recent plant inputs. These results not only illustrate the formidable complexity of mechanisms controlling C fluxes in soils but also suggest that the dual biological and physical effects of CO_2_ on primary productivity and global temperature have the potential to synergistically increase the mineralisation of existing soil C.

## Introduction

Atmospheric carbon dioxide (CO_2_) is both the primary source of carbon (C) for terrestrial photosynthetic organisms and a strong driver of the global climate (IPCC *et al*., 2007a,b). Atmospheric CO_2_ concentrations have risen by almost 80 ppm (ca. 24%) since 1959 and are now increasing at a rate of about 2 to 2.5 ppm per year (Tans & Keeling, 2014). Land temperatures in the Northern Hemisphere have been rising at a rate exceeding 0.3 °C per decade since 1979 (IPCC *et al*., 2007a,b). If recent trends continue, before the end of the century atmospheric CO_2_ concentrations will increase by over 50% and land temperatures in the Northern Hemisphere will rise by over 3 °C (IPCC *et al*., 2007b; Tans & Keeling, 2014).

More than 3000 Pg C is stored in soils, four times as much as is present in the atmosphere and about four times as much as in biomass (Sabine *et al*., 2004; IPCC *et al*., 2007a). Consequently, knowing whether atmospheric CO_2_ will increase soil C due to stimulation of plant productivity or decrease soil C due to temperature-driven increases in decomposition rates, is crucial to predictions of future climate (Davidson & Janssens, 2006; IPCC *et al*., 2007a,b; Trumbore & Czimczik, 2008; von Lützow & Kögel-Knabner, 2009; Conant *et al*., 2011). Belowground respiration (respiration due to microbial mineralisation of soil organic carbon (SOC), and respiration of recently fixed plant C by roots and rhizosphere microorganisms) accounts for up to a third of annual terrestrial and marine inputs of CO_2_ to the atmosphere (Boone *et al*., 1998; Sabine *et al*., 2004; IPCC *et al*., 2007a).

Due to the complexity of interactions between biosphere, atmosphere and climate, predictions of future climate change are only possible using mathematical models. To parameterise these models, there is a pressing need for a mechanistic understanding of SOC responses to increases in both atmospheric CO_2_ and temperature (Schmidt *et al*., 2011). However, after thousands of investigations our understanding of the mechanisms controlling the return of SOC to the atmosphere via microbial respiration remains poor (Davidson & Janssens, 2006; Trumbore & Czimczik, 2008; von Lützow & Kögel-Knabner, 2009; Conant *et al*., 2011; Schmidt *et al*., 2011).

Increases in soil temperature may accelerate losses of SOC due to effects of temperature on the reactions performed by soil microbes, which lead to more rapid mineralisation of SOC to CO_2_ (Davidson & Janssens, 2006; Trumbore & Czimczik, 2008; Conant *et al*., 2011). Conversely, elevated atmospheric CO_2_ may increase plant productivity, thereby increasing the rate of addition of new C to soils through larger roots and greater rhizodeposition (van Ginkel *et al*., 1997; Suter *et al*., 2002; Hill *et al*., 2007a; Phillips *et al*., 2009). However, inputs of relatively labile plant C to soils can also increase the rate of mineralisation of older SOC by rhizosphere priming (Dijkstra & Cheng, 2007; Fontaine *et al*., 2007; Kuzyakov, 2010; Schmidt *et al*., 2011; Hartley *et al*., 2012; Zhang *et al*., 2013). This has been suggested as an explanation for the fact that predicted increases in SOC due to elevated CO_2_ can often not be verified during experimental CO_2_ enrichment (Hoosbeek *et al*., 2004; van Groenigen *et al*., 2006; Kuzyakov, 2010). It has also been proposed that effects of atmospheric CO_2_ on soil temperature and CO_2_-driven increases in rhizosphere priming will have an additive effect on the loss of existing SOC to the atmosphere (Bardgett, 2011). However, despite very considerable research effort, both the individual and the combined effects of temperature and elevated CO_2_ on SOC remain uncertain (Davidson & Janssens, 2006; van Groenigen *et al*., 2006; Trumbore & Czimczik, 2008; Kuzyakov, 2010; Bardgett, 2011; Conant *et al*., 2011; Schmidt *et al*., 2011). This uncertainty arises largely from the difficulty of elucidating mechanisms in intact plant-soil systems with their complex collection of C fluxes. Belowground respiration is dependent to varying degrees upon a wide range of plant factors such as photosynthesis, plant C partitioning, root respiration, mycorrhizal colonisation, exudation and turnover, and microbial factors such as C substrate availability, C use efficiency, and community composition (Janssens *et al*., 2001; Kirschbaum, 2004; Pendall *et al*., 2004; Kuzyakov, 2006; Hill *et al*., 2007a,b; Hughes *et al*., 2008; Manzoni *et al*., 2012). All of these factors have some uncertainty in their responses to temperature and this is exacerbated by the fact that many plant and soil microbial processes frequently show some degree of thermal adaptation or acclimation to temperature change (Rovira, 1969; Gunn & Farrar, 1999; Covey-Crump *et al*., 2002; Pendall *et al*., 2004; Fang *et al*., 2005; Hill *et al*., 2007b; Luo, 2007; Boddy *et al*., 2008; von Lützow & Kögel-Knabner, 2009; Bergston *et al*., 2012; Manzoni *et al*., 2012; Craine *et al*., 2013; Hopkins *et al*., 2013; Tucker *et al*., 2013; Yin *et al*., 2013; Lefèrvre *et al*., 2014). Consequently, many investigations examining the effects of temperature on SOC mineralisation have been conducted by incubation of soils without the presence of living plants (Fang *et al*., 2005; Curiel Yuste *et al*., 2010; Conant *et al*., 2011; Hopkins *et al*., 2012). When in some investigations the magnitude of the response of belowground respiration to temperature has appeared to be enhanced by the presence of living roots, the difficulty of distinguishing between increases in SOC mineralisation and respiration of recently fixed root and rhizosphere C has hampered interpretation (Boone *et al*., 1998; Epron *et al*., 2001). Concurrent seasonal changes in soil temperature and plant C fixation under field conditions exacerbate problems (Epron *et al*., 2001; Högberg *et al*., 2001). We attempted to address this issue by applying a 3 °C increase in ambient soil temperature to established grass swards with living roots *in situ*. We compared the response of belowground respiration from these swards to soil temperature with that of soil without recent plant inputs. We used ^14^C dating of respired CO_2_ to aid separation of the response of root and rhizosphere respiration of recent C from that of microbial mineralisation of older SOC.

## Materials and methods

### Site location

Experiments were carried out on *Lolium perenne* L.-dominated grass swards at Bangor University Henfaes Experimental Station, Abergwyngregyn, Gwynedd, UK (53° 14′N, 4° 01′W). The mean annual rainfall is 1250 mm and the mean annual soil temperature at a soil depth of 10 cm is 11 °C. The soil is classified as a Eutric Cambisol (FAO) or Dystric Eutrudepts (US Soil Taxonomy) and is derived from Ordovician postglacial alluvial deposits. The site is well-drained and shows no indication of waterlogging. Prior to this experiment the site was permanent pasture for sheep grazing and we have no record of other land use. Over the last 50 years, this site has undergone an increase in air temperature of 0.2 °C per decade (measured 1959 to 2013; Figure S1).

### Grass swards

**Figure 1:**
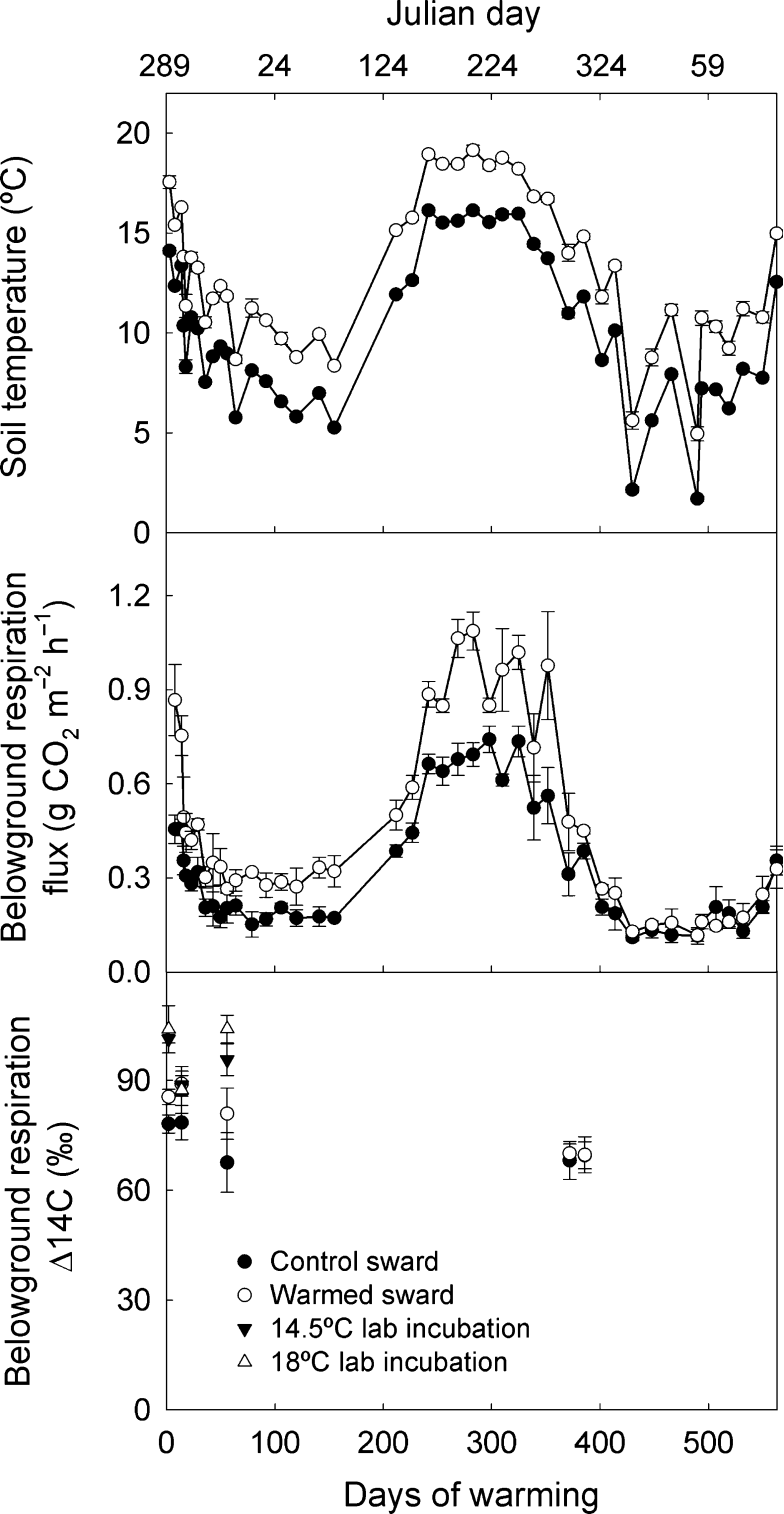
Soil temperature and belowground respiration in the field experiment with ^14^C content of CO_2_ respired in field and laboratory. Soil temperature and belowground respiration for control and warmed swards are shown in the upper and middle panels, respectively. Values for the ^14^C content of respired CO_2_ are shown on the lower panel. All values are mean ± SEM; *n *= 3.

Heating tape (RS Components, Corby, UK) was inserted in the soil of six 0.5 × 0.5 m portions of grass sward at a depth of 5 cm and at 5 cm intervals horizontally. To minimise disturbance, soil was cut with a knife and heating tape was pushed into the incision. A 4 cm long temperature probe was inserted to a depth of 7 cm between two sections of heating tape close to the centre of each plot. These probes were attached to RESOL DeltaSol Pro temperature differential regulators (RESOL, Hattingen, Germany). Three probes were used to determine ambient soil temperature (control plots) and three were used to measure the temperature in warmed plots. Polypropylene board was inserted into the soil around the plots to a depth of 20 cm to prevent lateral movement of CO_2_ from outside the treatment area. Swards were allowed to recover from disturbance for 6 weeks before the start of treatments. After 6 weeks, power was applied to the heating tape in three plots. The soil temperature of warmed plots was maintained at 3.0 ± 0.04 °C (mean ± SEM; *n *=* *49; Fig.1) above controls. To avoid overheating of soil and plants close to the heating tape and/or the generation of a temperature gradient, the current supplied to the heating tape was restricted to ca. 0.2 A (240 V). Measurements with a 2 mm diameter temperature probe from 0.5 to 2.5 cm from the tape could detect no temperature gradient. The treatment was maintained continuously for 80 weeks. During this period, swards were not cut or fertilised and grazing animals were excluded by fencing.

For CO_2_ flux measurement and capture, a 10 cm diameter circular portion at the centre of each plot was maintained without plant shoots by shading with opaque polypropylene tubs. Roots were allowed to grow in the soil underneath, so that CO_2_ respired by roots and soil microorganisms could be captured without contamination from shoot-derived CO_2_. Two 5 cm Rhizon soil solution samplers (Rhizosphere Research Products, Wageningen, the Netherlands) were inserted into each experimental plot at ca. 5 cm either side of shaded areas, at an angle of ca. 45° and to a depth of ca. 8 cm.

### Soil without plants

Soil was collected from three 0.75 m^2^ plots immediately adjacent to the experimental plots used for the field warming experiment. Prior to soil collection, plots had been covered with porous, opaque polypropylene matting for 15 months to ensure removal of all recent inputs of plant C. The matting excluded light but allowed water and gas exchange through it. Approximately 900 g DW soil was placed in each of six 1.7 l cylindrical polypropylene containers, packed to field bulk density (1.3 g DW cm^−3^) and incubated in the laboratory at 14.5 or 18 °C by submersion of containers in water baths. Prior to incubation, the containers of soil were allowed to recover from disturbance for 3 weeks at ambient outside temperature. Soil moisture was maintained at 0.5 g g^−1^ DW soil gravimetrically by additions of de-ionised water.

### Measurements

Soil temperature and CO_2_ efflux were measured in swards and soils without plants in the field for 80 and 48 weeks, respectively. Soil CO_2_ efflux was measured with an EGM-4 and SRC-1 soil respiration chamber (PP Systems, Hitchin, UK). Permanent collars were not inserted to allow free root growth under the measurement area. Soil temperature was measured using a temperature probe integrating over ca. 0–7 cm depth. Soil solution under grass swards was sampled on 20 occasions over the first 44 weeks of the warming treatment. Collected soil solution was analysed for dissolved organic C and total soluble N in a TOC-V-TN analyser (Shimadzu Corp., Kyoto, Japan), and NH_4_^+^ and NO_3_^−^ were analysed colorimetrically according to Mulvaney (1996) and Miranda *et al*. (2001), respectively. Total N not accounted for by inorganic forms of N was assumed to be dissolved organic N (DON). Each replicate was the mean of soil solution from the two Rhizon samplers in each plot. Plant biomass was sampled after 80 weeks of treatment by coring (38 mm diameter, 15 cm depth) roots or clipping shoots (0.04 m^2^ sward portions). Plant tissue and dry, root-free soil were analysed for total C and N content and *δ*^13^C in a PDZ Europa ANCA-GSL and PDZ Europa 20-20 (Sercon, Crewe, UK).

### Collection of CO_2_ for ^14^C dating

CO_2_ respired below ground in grass swards was collected for ^14^C dating after 2, 14, 56, 372 and 386 days of the warming treatment, and after 2, 14 and 56 day from incubated soils without plants. Portions of swards without shoots in the field, and containers of plant-free soil in the laboratory incubations, were covered with 10 cm diameter, 22 cm high, opaque, cylindrical polypropylene containers with 4 mm i.d. PVC tubing providing gas inlets and outlets. Containers over swards in the field were sealed by pushing them a few mm into the soil and those in the laboratory were sealed to soil containers with adhesive tape. CO_2_-free air was pumped through the containers until the CO_2_ concentration of air coming from the container fell to <5 ppm, after which time tubes were sealed with clamps. CO_2_ was allowed to accumulate for 24 h to avoid any influence of diurnal variation in the composition of respired CO_2_. After 24 h, the CO_2_ accumulated in the containers was pumped out of the containers and captured in zeolite molecular sieve according to Hardie *et al*. (2005). Following capture, CO_2_ was liberated by heating to 500 °C, cryogenically recaptured, converted to graphite by Fe/Zn reduction and analysed for ^14^C content by accelerator mass spectrometry at the Scottish Universities Environmental Research Centre (East Kilbride, UK).

### Calculations

*Q*_10_ values were calculated using a van't Hoff expression (Davidson *et al*., 2006). From combined plots of respiration against temperature curves of the form:

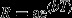
1 were fitted to data (Luo *et al*., 2001). Where *R* is respiration, *T* is temperature and *a* and *b* are fitted parameters. Q_10_s were calculated according to:

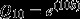
2 Δ^14^C of captured CO_2_ was calculated as:

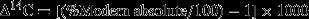
3

Making the assumption that all of the C had been fixed after the 1963 atmospheric bomb ^14^C peak, dates associated with Δ^14^C values were estimated from data for European atmospheric ^14^CO_2_ presented as the Jungfraujoch fit curve of Fig.1 in Levin *et al*. (2008).

Mean ages of SOC mineralised to CO_2_ from swards with living plants were calculated according to:

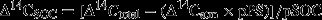
4 where Δ^14^C_SOC_ is the ^14^C content of mineralised SOC_,_ Δ^14^C_total_ is the measured ^14^C content of captured CO_2_, Δ^14^C_atm_ is the ^14^C content of the atmosphere at the time of measurement (current photosynthesis), pPS is the proportion of belowground respiration due to root and rhizosphere respiration and pSOC is the proportion of belowground respiration accounted for by SOC mineralisation. We use a Δ^14^C for atmospheric CO_2_ at the time of CO_2_ capture (2006–2007) of 55 ‰ (Levin *et al*., 2008).

Statistical analysis was by linear regression, *t*-test, repeated measures or oneway anova with Tukey post hoc test (SPSS v20; IBM, Armonk, NY, USA). Homogeneity of variance and normality were examined with Levene's test and Shapiro–Wilk test, respectively.

## Results

### Grass swards

**Figure 2:**
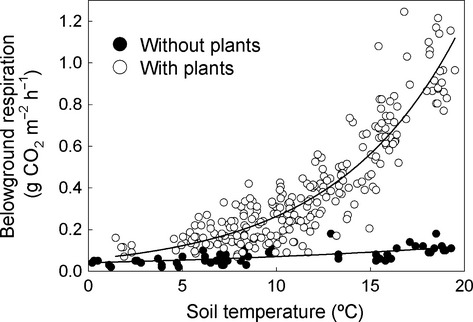
Response of belowground respiration to temperature in plots with and without plants. Values are individual measurements in the field for the entire 80 weeks of the experiment. Data from both warmed and control treatments of swards are included. Thus, seasonal changes in belowground respiration driven by photosynthesis are included where plants were present (open circles). The fitted line is: *y* = 0.0412e^(0.0535x)^; *Q*_10_ = e^(0.0535 × 10)^; *r*^2^ = 0.421; *n *= 58 for soil without plants (filled circles) and *y* = 0.0573e^(0.1524x)^; *Q*_10_ = e^(0.1524 × 10)^; *r*^2^ = 0.831; *n *= 252 for soil with plants.

Warming the soil under swards increased (*P *=* *0.02) the flux of belowground CO_2_ by a factor of 1.5 ± 0.04 (mean ± SEM; *n *=* *28; Fig.1) for 48 weeks. Although respiration eventually acclimated to the increase in soil temperature, over the 48 weeks when warming had an effect we estimate that warmed swards respired ca. 1.2 kg C m^−2^ and control plots respired ca. 0.83 kg C m^−2^ (calculated from the area under Fig.1). This indicates an overall *Q*_10_ due to experimental warming of 3.5. Assuming no treatment-induced alteration to plant phenology, this value should be independent of seasonal effects on plant productivity, which magnify the apparent response of belowground respiration to temperature when seasonality alters temperature and photosynthesis concurrently (Fig.2; *Q*_10_ = 4.6).

Over the first 2 weeks, warming increased the ^14^C content (Δ^14^C) of the respired CO_2_ by 9.0 ± 1.6 ‰ (mean ± SEM; *n *=* *2; *P *<* *0.04; Fig.1; details of individual analyses are presented in Supporting Information). We estimate that the CO_2_ respired from warmed swards had a mean age (relative to current photosynthetic C fixation) of about five or 6 years and that from control swards was about one or 2 years more recent. After 2 months, the ^14^C content of CO_2_ from warmed swards had fallen to that of control swards. The ^14^C content of CO_2_ from control swards did not change over the five occasions on which ^14^C was measured. There was no effect of warming on any other measured plant, soil or soil solution solute characteristic (Table1; Figure S2). Over all samples, dissolved organic C was weakly correlated with temperature, but this was probably largely driven by seasonal effects on plant productivity (*r*^2^* *=* *0.49; *P *<* *0.001; *n *=* *117; Figure S3).

**Table 1:** Soil and plant characteristics

Soil		
Without plants		
Total C (mg g^−1^ DW)	41 ± 0.7	
Total N (mg g^−1^ DW)	4.6 ± 0.04	
*δ*^13^C (‰)	−28.5 ± 0.05	
With plants	Control	Heated
Total C (mg g^−1^ DW)	47 ± 2	44 ± 2
Total N (mg g^−1^ DW)	4.9 ± 0.09	4.9 ± 0.1
*δ*^13^C (‰)	−28.5 ± 0.1	−28.7 ± 0.1
Soil solution		
Dissolved C (mg C l^−1^)	44 ± 2	41 ± 2
Total N (mg N l^−1^)	4.1 ± 0.3	7.3 ± 0.8
NO_3_^-^ (mg N l^−1^)	1.1 ± 0.2	2.8 ± 0.5
NH_4_^+^ (mg N l^−1^)	0.58 ± 0.07	0.59 ± 0.1
Dissolved organic N (mg N l^−1^)	2.3 ± 0.1	3.3 ± 0.3
Plants		
Root		
Biomass (kg DW m^−2^)	0.62 ± 0.08	0.51 ± 0.09
Total C (g g^−1^ DW)	0.39 ± 0.02	0.40 ± 0.01
Total N (mg g^−1^ DW)	14 ± 0.7	13 ± 0.3
*δ*^13^C (‰)	−30.3 ± 0.09	−30.6 ± 0.1
Shoot		
Biomass (kg DW m^−2^)	1.1 ± 0.1	1.4 ± 0.3
Total C (g g^−1^ DW)	0.43 ± 0.06	0.42 ± 0.5
Total N (mg g^−1^ DW)	14 ± 2	17 ± 1
*δ*^13^C (‰)	−29.8 ± 0.4	−30.1 ± 0.4
Collected CO_2_		
With plants *δ*^13^C (‰)	−27.4 ± 0.3	−26.6 ± 0.5
Without plants *δ*^13^C (‰)	−29.1 ± 0.2	−29.2 ± 0.1

C, N and *δ*^13^C for soil without plants are samples taken at the start of incubations. For soil without plants, Control indicates 14.5 °C and Heated indicates 18 °C incubation temperature. All values are mean ± SEM; *n *=* *3 except for soil solution solute concentrations where *n *=* *57 to 60, and *δ*^13^C of collected CO_2_ where *n *=* *15 and *n *=* *9 for soils with and without plants, respectively.

### Soils without plants

**Figure 3:**
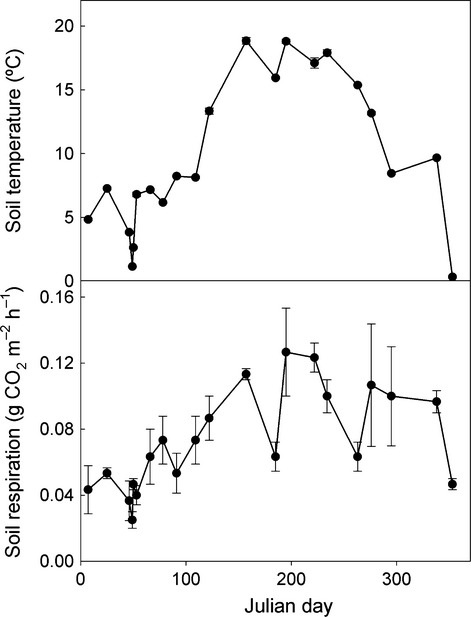
Seasonal variation in soil temperature and respiration in soils without plants. Values are mean ± SEM; *n *= 3.

Respiration from soil without plants had a relatively weak and variable response to seasonal changes in temperature (Q_10_ = 1.7; Fig.2) (we assume here that seasonal temperature change in the absence of plants was comparable to the experimental temperature alteration in swards). Similarly, a 3.5 °C difference in laboratory incubation temperature did not alter the ^14^C content of CO_2_ respired from this soil, which had a Δ^14^C suggesting a mean age of around 7 or 8 years (Fig.1). We estimate that under field conditions, the soil without recent plant inputs lost 0.174 kg C m^−2^ over 48 weeks (Fig.3).

## Discussion

During the first 48 weeks of treatment the 3 °C warming had a strong effect on below ground respiratory CO_2_ efflux from soils with plants. It is possible that the warming treatment caused some drying of soils. Relative to the effects of temperature, soil respiration frequently has low sensitivity to water content outwith extremes where availability of water or oxygen are limiting (Liu *et al*., 2002; Curiel Yuste *et al*., 2003; Xu *et al*., 2004). In our opinion, the free draining soil and frequent rainfall events throughout the year at the experimental site make it unlikely that such extremes were reached in grass swards of either treatment.

Belowground respiration is a composite of CO_2_ derived from root-dependent respiration (respiration from living roots and from microbial mineralisation of rhizodeposits) and SOC with a range of different ages and composition. This hampers the interpretation of experiments where respiration is measured with living plants *in situ*. The CO_2_ respired from warmed plots was also more enriched with ^14^C than that respired from control plots over the first 2 weeks of treatment. This ^14^C enrichment gives us confidence that the increase in below ground respiratory flux from warmed soils with plants was not due solely to an increase in root-dependent respiration of recent plant C inputs to the soil, but to a genuine increase in mineralisation of older SOC. The continued increase in CO_2_ flux with the same ^14^C signature indicates that the increase in SOC mineralisation due to the temperature treatment was sustained beyond the first 2 weeks when ^14^C enrichment of CO_2_ was different. Because the captured CO_2_ is a composite of CO_2_ respired from various ages of SOC, the CO_2_^14^C signature cannot distinguish a small increase in mineralisation of older SOC (e.g., 30 years old) from a larger increase in mineralisation of younger SOC (e.g., 10 years old). However, to estimate the mean age of the SOC mineralised in the presence of plants, it is necessary to make an estimate of the proportion of captured CO_2_ which can be attributed to this flux. Published values of the relative contributions of root and rhizosphere respiration and microbial respiration of older SOC to total belowground respiration are very variable (ranging from around 10% to 90% for root and rhizosphere respiration) and our results highlight why this is so (Epron *et al*., 2001; Hanson *et al*., 2000; Baggs, 2006; Kuzyakov, 2006; Koerber *et al*., 2010). Furthermore, we cannot be certain that the ratio of root-dependent respiration to SOC mineralisation remained constant between treatments and the responses of root and rhizomicrobial respiration, rhizodeposition and mineralisation of SOC to temperature are hard to predict (Rovira, 1969; Grayston *et al*., 1997; Boone *et al*., 1998; Gunn & Farrar, 1999; Atkin *et al*., 2000; Uselman *et al*., 2000; Covey-Crump *et al*., 2002; Hill *et al*., 2007b; Boddy *et al*., 2008; von Lützow & Kögel-Knabner, 2009).

Assuming a conservative and constant 50% contribution of recent C to the total CO_2_ flux in both treatments, the mean age of SOC mineralised to CO_2_ in control swards and warmed swards after the first 2 weeks was around 8 years old, and that from warmed swards within the first 2 weeks was about 10 years old. This suggests that 0.415 kg SOC m^−2^ with a mean age of ca. 8 years was mineralised to CO_2_ over 48 weeks in control swards, and that the increase in loss of SOC with a mean age of ca. 8 years or more due to the 3 °C increase in soil temperature was 0.185 kg C m^−2^. Thus, assuming that the ^14^C content of CO_2_ captured during laboratory incubations was representative of that respired in the field, losses of SOC with a mean age of ca. 8 years from soils without plants were under half of those from control swards, and less than the difference induced by a 3 °C increase in sward soil temperature. Furthermore, our field-measured *Q*_10_ of 1.7 suggests that a 3 °C increase in temperature would only increase SOC mineralisation in unplanted soils by 0.079 kg C m^−2^, less than half the increase in soils with plants.

Although, the presence of SOC with an age younger than 15 months in unplanted soils would probably decrease the magnitude of the difference in respiratory fluxes between planted and plant-free soils, it would inevitably decrease the age of the CO_2_ respired from the soil without plants. Similarly, if the contribution of recent root and rhizosphere C to belowground CO_2_ fluxes was greater than our assumed 50%, then the increase in SOC mineralisation due to roots and/or warming was less than we have estimated, but the mean age of the SOC mineralised was greater (e.g. a 70% contribution of root and rhizosphere respiration would indicate a warming-induced increase in SOC mineralisation CO_2_ flux of 0.11 kg C m^−2^ over 48 weeks with a mean age of ca. 15 years whereas a 30% contribution would indicate a flux of 0.26 kg C m^−2^ with a mean age of ca. 6 years). Thus, although we are not able to estimate the age or flux of the lost SOC with great precision, it is clear that the presence of living roots both accelerated SOC mineralisation and increased the magnitude of the response of SOC mineralisation to increased soil temperature. This interaction between living roots, SOC mineralisation and temperature suggests that the physical effects of atmospheric CO_2_ on global temperatures and biological effects on plant productivity have the potential to synergistically increase the mineralisation of existing SOC. It also highlights the formidable barriers encountered when trying to understand or model the mechanisms controlling C fluxes in ecosystems.

Many (but not all) investigations using experimental warming have reported some form of acclimation or thermal adaptation of below ground respiration to temperature increase, although the duration over which an effect of temperature can be measured varies (Luo *et al*., 2001; Melillo *et al*., 2002; Kirschbaum, 2004; Hartley *et al*., 2008; Craine *et al*., 2013). The exact cause of this acclimation is unknown, but microbial physiology, changes to soil microbial communities and C substrate availability are all implicated (Kirschbaum, 2004; Bradford *et al*., 2008; Tucker *et al*., 2013). We are unable to determine the mechanism or mechanisms driving the increase in SOC mineralisation or subsequent acclimation in our investigation and a range of possibilities exist. It is possible that a combination of warming and root priming increased the mineralisation of SOC with a particular age with acclimation occurring due to subsequent lower availability of this respiratory substrate. Alternatively, warming and roots may have increased mineralisation of SOC more widely via increased microbial activity or perhaps reduced C use efficiency with later acclimation of microbial physiology or changes to the microbial community structure. It may be that no single mechanism was responsible.

The acclimation of the response of SOC mineralisation to temperature within a year in our investigation may indicate that future increases in temperature will not lead to catastrophic positive feedback on climate due to losses of SOC. If this is the case, a 3 °C temperature increase will deliver only a modest 1% increase in atmospheric CO_2_ (relative to current concentration) due to the mineralisation of C stored in grassland soils (Sabine *et al*., 2004). However, experimental manipulation can never fully simulate climate change and it is not currently clear whether acclimation of SOC mineralisation to temperature will remain under the influence of the dual physical and biological mechanisms for positive feedback on atmospheric CO_2_. Investigations in forest ecosystems indicate that synergy between plant productivity and temperature accelerates SOC loss more widely than grassland and it therefore seems probable that this process could be universal in plant-soil systems (Boone *et al*., 1998; Epron *et al*., 2001; Curiel Yuste *et al*., 2010). If this is the case, global loss of existing soil C to the atmosphere as atmospheric CO_2_ increases, and consequent positive feedback, is likely to be considerable.

## Supporting Information

**Figure S1.** Mean daily air temperature at Bangor University's Henfaes research station between 1959 and 2013. **Figure S2.** Soil solution solute concentrations in experimental grass swards. **Figure S3.** Relationship between soil solution dissolved organic carbon (DOC) concentration and soil temperature in grass swards. **Table S1.** Details of individual ^14^C analyses.
