# Supplementary material for: Living roots magnify the response of soil organic carbon decomposition to temperature in temperate grassland
Source: Glob Chang Biol. 2014 Dec 23;21(3):1368–75. doi: 10.1111/gcb.12784 (PMC4365897; doi:10.1111/gcb.12784)

**Supporting Information Table 1** Details of individual ^14^C analyses

| **Soil** | **Treatment** | **Day of treatment** | **Laboratory code** | **% modern absolute ± 1σ** | **Δ^14^C**  **(‰)** | **δ^13^C_VPDB_ (‰)** |
| --- | --- | --- | --- | --- | --- | --- |
| No plants | 14.5 °C | 2 | SUERC-13346 | 109.95 ± 0.49 | 99.5 | -29.9 |
|  |  |  | SUERC-13348 | 110.33 ± 0.48 | 103.3 | -28.5 |
|  |  |  | SUERC-13352 | 110.20 ± 0.48 | 102.0 | -28.4 |
|  |  | 14 | SUERC-13364 | 108.41 ± 0.52 | 84.1 | -28.9 |
|  |  |  | SUERC-13365 | 109.29 ± 0.48 | 92.9 | -28.7 |
|  |  |  | SUERC-13366 | 109.00 ± 0.48 | 90.0 | -29.3 |
|  |  | 56 | SUERC-13375 | 110.01 ± 0.48 | 100.1 | -29.3 |
|  |  |  | SUERC-13376 | 110.04 ± 0.48 | 100.4 | -28.8 |
|  |  |  | SUERC-13377 | 108.72 ± 0.50 | 87.2 | -29.7 |
|  | 18 °C | 2 | SUERC-13345 | 111.69 ± 0.49 | 116.9 | -28.7 |
|  |  |  | SUERC-13347 | 109.97 ± 0.48 | 99.7 | -28.8 |
|  |  |  | SUERC-13351 | 109.60 ± 0.48 | 96.0 | -28.8 |
|  |  | 14 | SUERC-13361 | 109.68 ± 0.48 | 96.8 | -29.7 |
|  |  |  | SUERC-13362 | 109.09 ± 0.48 | 90.9 | -29.0 |
|  |  |  | SUERC-13363 | 107.53 ± 0.51 | 75.3 | -28.9 |
|  |  | 56 | SUERC-13378 | 111.07 ± 0.49 | 110.7 | -29.5 |
|  |  |  | SUERC-13381 | 110.47 ± 0.48 | 104.7 | -29.6 |
|  |  |  | SUERC-13382 | 109.75 ± 0.48 | 97.5 | -29.5 |
| Sward | Control | 2 | SUERC-12613 | 108.99 ± 0.47 | 89.9 | -27.9 |
|  |  |  | SUERC-12615 | 107.80 ± 0.47 | 78.0 | -27.7 |
|  |  |  | SUERC-12617 | 107.43 ± 0.47 | 74.3 | -26.8 |
|  |  | 14 | SUERC-13353 | 106.96 ± 0.47 | 69.6 | -28.6 |
|  |  |  | SUERC-13357 | 108.17 ± 0.47 | 81.7 | -28.7 |
|  |  |  | SUERC-13355 | 108.49 ± 0.48 | 84.9 | -27.5 |
|  |  | 56 | SUERC-13367 | 106.43 ± 0.47 | 64.3 | -28.4 |
|  |  |  | SUERC-13371 | 105.58 ± 0.46 | 55.8 | -29.3 |
|  |  |  | SUERC-13373 | 108.33 ± 0.48 | 83.3 | -28.1 |
|  |  | 372 | SUERC-16789 | 107.79 ± 0.47 | 77.9 | -26.2 |
|  |  |  | SUERC-16791 | 106.76 ± 0.50 | 67.6 | -25.6 |
|  |  |  | SUERC-16793 | 106.01 ± 0.49 | 60.1 | -24.9 |
|  |  | 386 | SUERC-16797 | 106.99 ± 0.50 | 69.9 | -27.9 |
|  |  |  | SUERC-16799 | 107.63 ± 0.50 | 76.3 | -27.0 |
|  |  |  | SUERC-16801 | 106.35 ± 0.47 | 63.5 | -25.8 |
|  | Warmed | 2 | SUERC-12614 | 108.42 ± 0.47 | 84.2 | -28.0 |
|  |  |  | SUERC-12616 | 108.98 ± 0.48 | 89.8 | -26.8 |
|  |  |  | SUERC-12618 | 109.03 ± 0.48 | 90.3 | -26.9 |
|  |  | 14 | SUERC-13356 | 108.94 ± 0.48 | 89.4 | -28.8 |
|  |  |  | SUERC-13354 | 109.52 ± 0.48 | 95.2 | -27.8 |
|  |  |  | SUERC-13358 | 108.34 ± 0.49 | 83.4 | -27.2 |
|  |  | 56 | SUERC-13368 | 107.77 ± 0.47 | 77.7 | -29.4 |
|  |  |  | SUERC-13372 | 109.47 ± 0.48 | 94.7 | -29.1 |
|  |  |  | SUERC-13374 | 107.12 ± 0.47 | 71.2 | -29.1 |
|  |  | 372 | SUERC-16790 | 107.53 ± 0.50 | 75.3 | -23.7 |
|  |  |  | SUERC-16792 | 106.91 ± 0.50 | 69.1 | -24.5 |
|  |  |  | SUERC-16794 | 106.69 ± 0.50 | 66.9 | -23.9 |
|  |  | 386 | SUERC-16798 | 107.97 ± 0.51 | 79.7 | -24.7 |
|  |  |  | SUERC-16800 | 106.34 ± 0.50 | 63.4 | -24.4 |
|  |  |  | SUERC-16802 | 106.71 ± 0.48 | 67.1 | -25.2 |

**Supporting Information Figure 1:** Mean daily air temperature at Bangor University’s Henfaes research station between 1959 and 2013. The dashed line represents the 1959 mean and the solid line is linear regression showing long term trend (1°C over the last 50 years).


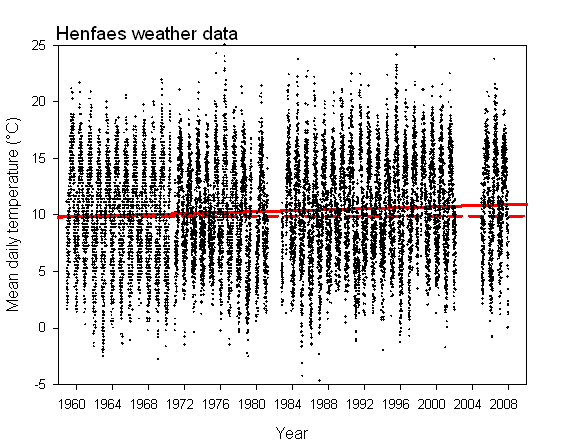


**Supporting Information Figure 2:** Soil solution solute concentrations in experimental grass swards. Values are mean ± SEM; *n*=3.

**Supporting Information Figure 3:** Relationship between soil solution dissolved organic carbon (DOC) concentration and soil temperature in grass swards. Values are individual samples from both treatments.


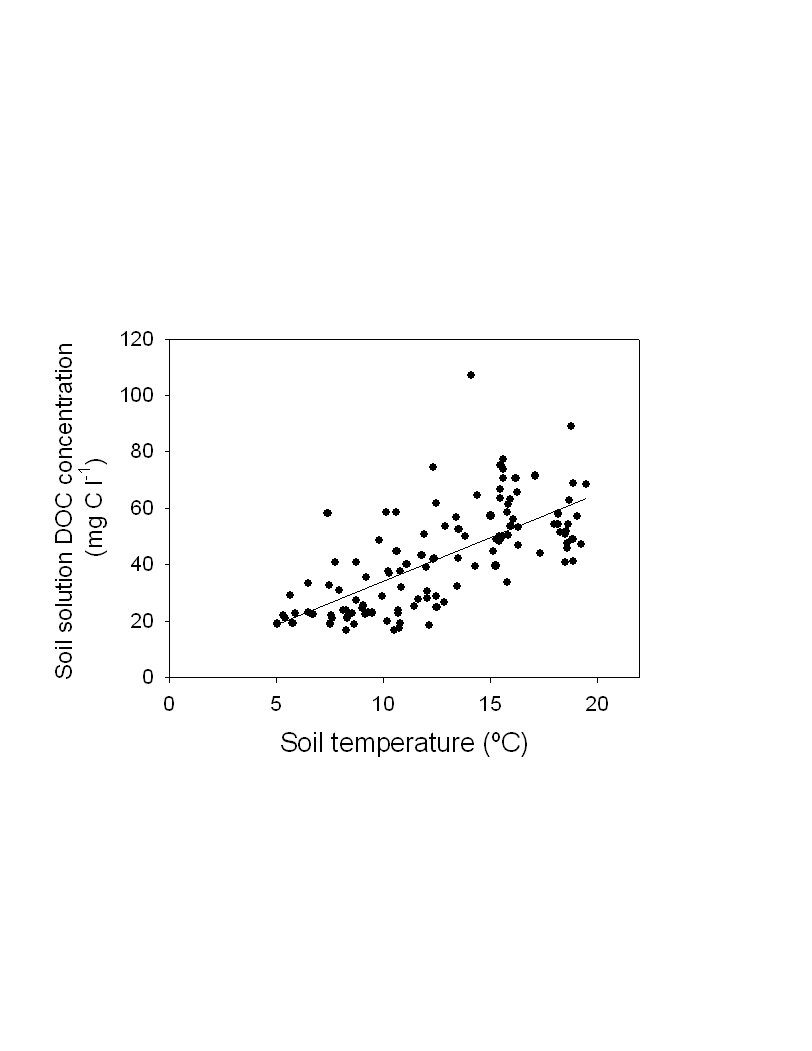

Supplement: Supplementary file 1 — Figure S1. Mean daily air temperature at Bangor University's Henfaes research station between 1959 and 2013. Figure S2. Soil solution solute concentrations in experimental grass swards. Figure S3. Relationship between soil solution dissolved organic carbon (DOC) concentration and soil temperature in grass swards. Table S1. Details of individual 14C analyses. [file gcb0021-1368-sd1.docx]
